# Association of Coronary Plaque With Low-Density Lipoprotein Cholesterol Levels and Rates of Cardiovascular Disease Events Among Symptomatic Adults

**DOI:** 10.1001/jamanetworkopen.2021.48139

**Published:** 2022-02-11

**Authors:** Martin Bødtker Mortensen, Miguel Caínzos-Achirica, Flemming Hald Steffensen, Hans Erik Bøtker, Jesper Møller Jensen, Niels Peter Rønnow Sand, Michael Maeng, Jens Meldgaard Bruun, Michael J. Blaha, Henrik Toft Sørensen, Manan Pareek, Khurram Nasir, Bjarne L. Nørgaard

**Affiliations:** 1 Department of Cardiology, Aarhus University Hospital, Aarhus, Denmark; 2 Johns Hopkins Ciccarone Center for the Prevention of Cardiovascular Disease, Johns Hopkins University School of Medicine, Baltimore, Maryland; 3 Division of Cardiovascular Prevention and Wellness, Department of Cardiology, Houston Methodist DeBakey Heart & Vascular Center, Houston, Texas; 4 Center for Outcomes Research, Houston Methodist, Houston, Texas; 5 Welch Center for Prevention, Epidemiology and Clinical Research, The Johns Hopkins University, Baltimore, Maryland; 6 Department of Cardiology, Lillebaelt Hospital-Vejle, Vejle, Denmark; 7 Department of Cardiology, University Hospital of Southwest Jutland and Institute of Regional Health Research, University of Southern Denmark, Esbjerg, Denmark; 8 Steno Diabetes Center Aarhus, Aarhus University Hospital, Aarhus, Denmark; 9 Department of Clinical Epidemiology, Aarhus University Hospital, Aarhus, Denmark; 10 Department of Internal Medicine, Yale New Haven Hospital, Yale University School of Medicine, New Haven, Connecticut

## Abstract

**Importance:**

Atherosclerosis burden and coronary artery calcium (CAC) are associated with the risk for atherosclerotic cardiovascular disease (ASCVD) events, with absence of plaque and CAC indicating low risk. Whether this is true in patients with elevated levels of low-density lipoprotein cholesterol (LDL-C) is not known. Specifically, a high prevalence of noncalcified plaque might signal high risk.

**Objective:**

To determine the prevalence of noncalcified and calcified plaque in symptomatic adults and assess its association with cardiovascular events across the LDL-C spectrum.

**Design, Setting, and Participants:**

This cohort study included symptomatic patients undergoing coronary computed tomographic angiography from January 1, 2008, to December 31, 2017, from the seminational Western Denmark Heart Registry. Follow-up was completed on July 6, 2018. Data were analyzed from April 2 to December 2, 2021.

**Exposures:**

Prevalence of calcified and noncalcified plaque according to LDL-C strata of less than 77, 77 to 112, 113 to 154, 155 to 189, and at least 190 mg/dL. Severity of coronary artery disease was categorized using CAC scores of 0, 1 to 99, and ≥100, where higher numbers indicate greater CAC burden.

**Main Outcomes and Measures:**

Atherosclerotic cardiovascular disease events (myocardial infarction and stroke) and death.

**Results:**

A total of 23 143 patients with a median age of 58 (IQR, 50-65) years (12 857 [55.6%] women) were included in the analysis. During median follow-up of 4.2 (IQR, 2.3-6.1) years, 1029 ASCVD and death events occurred. Across all LDL-C strata, absence of CAC was a prevalent finding (ranging from 438 of 948 [46.2%] in patients with LDL-C levels of at least 190 mg/dL to 4370 of 7964 [54.9%] in patients with LDL-C levels of 77-112 mg/dL) and associated with no detectable plaque in most patients, ranging from 338 of 438 (77.2%) in those with LDL-C levels of at least 190 mg/dL to 1067 of 1204 (88.6%) in those with LDL-C levels of less than 77 mg/dL. In all LDL-C groups, absence of CAC was associated with low rates of ASCVD and death (6.3 [95% CI, 5.6-7.0] per 1000 person-years), with increasing rates in patients with CAC scores of 1 to 99 (11.1 [95% CI, 10.0-12.5] per 1000 person-years) and CAC scores of at least 100 (21.9 [95% CI, 19.9-24.4] per 1000 person-years). Among those with CAC scores of 0, the event rate per 1000 person-years was 6.3 (95% CI, 5.6-7.0) in the overall population compared with 6.9 (95% CI, 4.0-11.9) in those with LDL-C levels of at least 190 mg/dL. Across all LDL-C strata, rates were similar and low in those with CAC scores of 0, regardless of whether they had no plaque or purely noncalcified plaque.

**Conclusions and Relevance:**

The findings of this cohort study suggest that in symptomatic patients with severely elevated LDL-C levels of at least 190 mg/dL who are universally considered to be at high risk by guidelines, absence of calcified and noncalcified plaque on coronary computed tomographic angiography was associated with low risk for ASCVD events. These results further suggest that atherosclerosis burden, including CAC, can be used to individualize treatment intensity in patients with severely elevated LDL-C levels.

## Key Points

**Question:**

What is the prevalence of coronary plaque, and is it associated with rates of cardiovascular events in patients with severely elevated low-density lipoprotein cholesterol (LDL-C) levels (≥190 mg/dL) who are universally considered to be at high risk?

**Findings:**

In this cohort study of 23 143 symptomatic patients, absence of coronary artery calcium (CAC) and noncalcified plaque was a prevalent finding among those with severely elevated LDL-C levels. Across the LDL-C spectrum, absence of CAC was associated with low rates of atherosclerotic cardiovascular disease and death, with increasing rates in patients with greater CAC burden.

**Meaning:**

These findings suggest that atherosclerosis burden, including assessment of CAC, can be used to individualize treatment intensity by identifying patients who are at low risk despite having severely elevated LDL-C levels.

## Teaser

This cohort study of symptomatic patients undergoing coronary computed tomographic angiography assesses the prevalence of calcified and noncalcified plaque and the association of these phenotypes with cardiovascular events across the spectra of low-density lipoprotein cholesterol levels.

## Introduction

US and European guidelines for the management of dyslipidemias in the prevention of atherosclerotic cardiovascular disease (ASCVD) provide strong recommendations (ie, class IA) for treating all patients with severe hypercholesterolemia (low-density lipoprotein cholesterol [LDL-C] levels ≥190 mg/dL) (to convert to mmol/L, multiply by 0.0259) with statins.^1,2^ Specifically, the 2019 dyslipidemia guidelines from the European Society of Cardiology universally classify patients with LDL-C levels of greater than 190 mg/dL as high-risk individuals, with an LDL-C treatment goal of less than 70 mg/dL. To achieve such a low LDL-C level in patients with severely elevated baseline levels, other therapies to lower lipid levels, such as ezetimibe and proprotein convertase subtilisin/kexin type 9 inhibitors, are needed despite use of maximally tolerated statin therapy.^1,2^

Recent studies in small populations undergoing primary prevention^3,4,5^ have suggested that a sizeable proportion of persons with severe hypercholesterolemia are resilient to developing calcified coronary plaque, as identified by a coronary artery calcium (CAC) score of 0. For example, in a study of 246 participants with LDL-C levels of at least 190 mg/dL from the Multi-Ethnic Study of Atherosclerosis,^4^ a CAC score of 0 was present in 37% of individuals, whereas in a meta-analysis of 1176 patients with genetically verified familial hypercholesterolemia,^5^ the prevalence of CAC scores of 0 was 45%. Importantly, CAC burden has been shown to stratify risk among various other lipid disorders, allowing for the identification of individuals with lowest and highest event rates.^6^

Despite these preliminary findings, concerns regarding a potentially high prevalence of noncalcified plaque in individuals with severe dyslipidemias (ie, clinical familial hypercholesterolemia) associated with very high LDL-C levels of at least 190 mg/dL remain, despite a CAC score of 0.^7^ Indeed, the European Atherosclerosis Society familial hypercholesterolemia consensus report states that “there would likely be diffuse, noncalcified plaques in such individuals.”^7^^(p3485)^ This has prevented exploring the potential value of using plaque burden and CAC for risk stratification, particularly for “derisking” (ie, lowering classification of risk) purposes, and of using personalized LDL-C management in these patients.^8^ Indeed, studies on the real-world prevalence and prognostic implications of noncalcified coronary atherosclerosis in persons with LDL-C levels of at least 190 mg/dL are scarce.^5^

Thus, the present study had 2 goals. The first goal was to describe, in a large, carefully phenotyped cohort of symptomatic patients undergoing coronary computed tomographic angiography (CCTA) assessment, the prevalence of calcified and noncalcified plaque. The second goal was to describe the association of these phenotypes with cardiovascular events across the following 5 LDL-C groups: levels of less than 77, 77 to 112, 113 to 154, 155 to 189, and at least 190 mg/dL.

## Methods

### Setting

In this cohort study, we used data from the Western Denmark Heart Registry (WDHR), a seminational ongoing longitudinal health registry.^9,10^ The WDHR includes all adult patients in Western Denmark who are referred for cardiac intervention (ie, coronary angiography, cardiac surgery). The WDHR sites use uniform data collection methods prespecified in the electronic WDHR data entry form. Data regarding smoking, hypertension, and diabetes are collected and reported by the treating physicians. The quality of WDHR data has been previously audited and validated.^9,10^ We followed the Strengthening the Reporting of Observational Studies in Epidemiology (STROBE) reporting guideline. The study was approved by the Danish Data Protection Agency. Because the data included were retrospective and deidentified, the regional ethics committee waived the requirement for individual consent.

The Danish National Health Service provides universal tax-supported health care. A Civil Personal Registration number is assigned to each Danish citizen, allowing linkage of the WDHR data to multiple national registries.^11^ Information on blood test measurements, including cholesterol levels, is available from the Clinical Laboratory Information System research database, which collects results from routine tests performed by hospitals, general clinicians, and specialists for analysis at hospital-based laboratories.^12^ The Danish National Patient Registry contains information on dates of admission to and discharge from all Danish hospitals and outpatient specialist clinics, using *International Classification of Diseases and Related Health Problems, Tenth Revision*, codes.^13^ The National Health Service Prescription Database contains information on all reimbursable prescriptions redeemed by Danish residents.^14^

### Study Population

We included all adult individuals (18 years or older) from the WDHR undergoing CCTA from January 1, 2008, to December 31, 2017, owing to symptoms suggestive of coronary artery disease. Exclusion criteria were known coronary artery disease at the time of the CCTA (either prior myocardial infarction or coronary revascularization) and missing information on pretest LDL-C level.

Coronary computed tomographic angiography was performed using different scanner platforms (Siemens AG, Toshiba Corporation, and Koninklijke Philips NV), all of which had at least 64 detector rows. Full details on CCTA acquisition in the WDHR has been described previously.^9,15^ Experienced local cardiologists (including F.H.S., J.M.J., N.P.R.S., and B.L.N.) analyzed all CCTA findings and reported the results to the WDHR. Information on total and high-density lipoprotein cholesterol levels was used if obtained as many as 5 years before the CCTA. If multiple measurements were available, the values closest to the date of the CCTA were included in the analyses. Information on statin use was obtained before and after the CCTA procedure date.

### Coronary Artery Disease Classification

We categorized coronary artery disease plaques into noncalcified and calcified plaques. Severity of coronary artery disease was categorized according to the presence vs absence of obstructive disease as well as based on the calcified atherosclerotic burden (CAC scores of 0, 1-99, and ≥100, where higher numbers indicate greater CAC burden). In the classification based on presence of obstructive disease, we defined the following groups: no coronary artery disease (0% luminal stenosis and absence of any plaque), nonobstructive coronary artery disease (any plaque and <50% luminal stenosis), and obstructive coronary artery disease (>50% luminal stenosis).

### Event Ascertainment and Study Outcomes

We defined a main composite end point of myocardial infarction, ischemic stroke, and all-cause death occurring more than 90 days after a CCTA until the first occurrence of any of these events or July 6, 2018, whichever occurred first. Secondary analyses were restricted to myocardial infarction and ischemic stroke.

### Statistical Analysis

Data were analyzed from April 2 to December 2, 2021. Baseline categorical variables were summarized using absolute number and proportion, and continuous variables were described using median (IQR). The prevalence of calcified coronary atherosclerotic plaque was assessed overall as well as by baseline LDL-C levels.

The incidence of outcomes was determined using crude event rates per 1000 person-years. These were calculated overall as well as by the LDL-C and plaque burden categories described above. Kaplan-Meier cumulative incidence curves were used to compare events among individuals with various degrees of baseline CAC burdens and LDL-C levels. Log-rank tests were used to formally compare these groups.

Cox proportional hazards regression models were used to assess the associations between baseline CAC burden and incident events during follow-up across LDL-C strata. Models were adjusted for age, sex, smoking status, body mass index, statin use at baseline, aspirin use at baseline, and post-CCTA statin use.

There were no losses to follow-up—that is, we accounted for each individual at all times until occurrence of an event, emigration, or end of follow-up. Two-sided *P* < .05 indicated statistical significance. Analyses were performed using Stata, version 15 (StataCorp LLC).

## Results

The study included 23 143 patients with a median age of 58 (IQR, 50-65) years, 10 286 (44.4%) men, and 12 857 (55.6%) women (Table 1). Race and ethnicity data were not collected. The prevalence of current smoking was 4768 (20.6%); hypertension, 10 714 (46.3%); and diabetes, 1996 (8.6%). In total, 2430 (10.5%) had LDL-C levels of less than 77 mg/dL; 7964 (34.4%), 77 to 112 mg/dL; 8409 (36.3%), 113-154 mg/dL; 3392 (14.7%), 155 to 189 mg/dL; and 948 (4.1%), at least 190 mg/dL. During a median of 4.2 (IQR, 2.3-6.1) years of follow-up, 1029 patients experienced a first event (myocardial infarction [n = 219], stroke [n = 299], or death [n = 511]).

**Table 1.** Baseline Characteristics of the Study Population

Characteristic	Patient group^a^					
	Overall	LDL-C level, mg/dL				
		<77	77-112	113-154	155-189	≥190
No. (%) of patients	23 143 (100)	2430 (10.5)	7964 (34.4)	8409 (36.3)	3392 (14.7)	948 (4.1)
Age, median (IQR), y	58 (50-65)	59 (51-67)	57 (49-65)	57 (50-65)	58 (51-65)	57 (50-64)
Sex						
Women	12 857 (55.6)	1343 (55.3)	4547 (57.1)	4538 (54.0)	1887 (55.6)	542 (57.2)
Men	10 286 (44.4)	1087 (44.7)	3417 (42.9)	3871 (46.0)	1505 (44.4)	406 (42.8)
Family history of CHD	10 447 (45.1)	1049 (43.2)	3659 (45.9)	3683 (43.8)	1576 (46.5)	480 (50.6)
BMI, median (IQR)	26 (24-29)	27 (24-29)	26 (23-29)	26 (24-29)	26 (24-29)	27 (24-29)
Tobacco use						
Current	4768 (20.6)	511 (21.0)	1638 (20.6)	1642 (19.5)	731 (21.5)	246 (26.0)
Former	7577 (32.7)	817 (33.6)	2597 (32.6)	2728 (32.4)	1135 (33.5)	300 (31.6)
Hypertension	10 714 (46.3)	1582 (65.1)	3963 (49.8)	3494 (41.6)	1343 (39.6)	332 (35.0)
Diabetes	1996 (8.6)	647 (26.6)	807 (10.1)	378 (4.5)	122 (3.6)	42 (4.4)
Cholesterol level, median (IQR), mg/dL						
LDL-C	120 (93-143)	66 (54-69)	97 (89-104)	132 (123-139)	166 (159-174)	205 (81-216)
HDL-C	54 (45-70)	54 (43-70)	58 (46-70)	54 (46-66)	54 (46-66)	50 (43-62)
Symptoms						
Chest pain^b^						
Typical	2698 (12.6)	301 (13.3)	924 (12.6)	980 (12.5)	385 (12.2)	108 (12.3)
Atypical	12 584 (58.6)	1299 (57.5)	4297 (58.4)	4571 (58.5)	1886 (59.5)	531 (60.3)
Unspecified	4759 (22.1)	505 (22.3)	1649 (22.4)	1743 (22.3)	689 (21.7)	173 (19.7)
Dyspnea^b^	1452 (6.8)	156 (6.9)	491 (6.7)	527 (6.7)	210 (6.6)	68 (7.7)
Medication use at baseline						
Statin^c^	6438 (27.8)	1464 (60.2)	2790 (35.0)	1253 (14.9)	604 (17.8)	327 (34.5)
Aspirin^d^	3363 (14.5)	728 (30.0)	1382 (17.4)	845 (10.0)	326 (9.6)	82 (8.6)
Medication use at >1 y after CCTA						
Statin	8923 (38.6)	1267 (52.1)	3013 (37.8)	2727 (32.4)	1390 (41.0)	526 (55.5)
Aspirin	5910 (25.5)	763 (31.4)	2036 (25.6)	2020 (24.0)	815 (24.0)	276 (29.1)

Abbreviations: BMI, body mass index (calculated as weight in kilograms divided by height in meters squared); CCTA, coronary computed tomographic angiography; CHD, coronary heart disease; HDL-C, high-density lipoprotein cholesterol; LDL-C, low-density lipoprotein cholesterol.

SI conversion factor: To convert HDL-C and LDL-C to mmol/L, multiply by 0.0259.

^a^ Unless otherwise indicated, data are expressed as number (%) of patients.

^b^ Owing to missing data, numbers may not sum to column headings.

^c^ Indicates redemption of statin prescriptions from pharmacies at least 2 times before CCTA.

^d^ Indicates redemption of aspirin prescriptions from pharmacies at least 2 times before CCTA.

### Prevalence of Atherosclerotic Plaque Across LDL-C Strata

In the overall population, a total of 10 708 patients (46.3%) had no detectable plaque (neither calcified nor noncalcified plaque), whereas 12 435 (53.7%) had detectable plaque. Stratified by presence of calcified plaque, 12 341 patients (53.3%) had CAC scores of 0; 6282 (27.1%), 1 to 99; and 4520 (19.5%), at least 100 (Table 2). Among those with CAC scores of 0, 10 708 (86.8%) had no detectable plaque, whereas 1633 (13.2%) had noncalcified plaque, including 711 (5.8%) with obstructive coronary artery disease.

**Table 2.** Prevalence of Calcified and Noncalcified Coronary Atherosclerotic Plaque

Baseline LDL-C level by CAC score^a^	No./total No. (%) of patients			
	Prevalence	CCTA finding		
		No plaque	Plaque	
			Nonobstructive	Obstructive
Overall				
0	12 341/23 143 (53.3)	10 708/12 341 (86.8)	922/12 341 (7.4)	711/12 341 (5.8)
1-99	6282/23 143 (27.1)	NA	4765/6282 (75.9)	1517/6282 (24.1)
≥100	4520/23 143 (19.5)	NA	1793/4520 (39.7)	2727/4520 (60.3)
<77 mg/dL				
0	1204/2430 (49.5)	1067/1204 (88.6)	81/1204 (6.7)	56/1204 (4.7)
1-99	619/2430 (25.5)	NA	492/619 (79.5)	127/619 (20.5)
≥100	607/2430 (25.0)	NA	256/607 (42.2)	351/607 (57.8)
77-112 mg/dL				
0	4370/7964 (54.9)	3865/4370 (88.4)	277/3865 (7.2)	228/3865 (5.9)
1-99	2081/7964 (26.1)	NA	1586/2081 (76.2)	495/2081 (23.8)
≥100	1513/7964 (19.0)	NA	613/1513 (40.5)	900/1513 (59.5)
113-154 mg/dL				
0	4620/8409 (54.9)	3981/4620 (86.2)	346/4620 (7.5)	293/4620 (6.3)
1-99	2261/8409 (26.9)	NA	1744/2261 (77.1)	517/2261 (22.9)
≥100	1528/8409 (18.2)	NA	612/1528 (40.1)	916/1528 (59.9)
155-189 mg/dL				
0	1709/3392 (50.4)	1457/1709 (85.3)	160/1709 (9.4)	92/1709 (5.4)
1-99	1040/3392 (30.7)	NA	757/1040 (72.8)	283/1040 (27.2)
≥100	643/3392 (19.0)	NA	235/643 (36.5)	408/643 (63.5)
≥190 mg/dL				
0	438/948 (46.2)	338/438 (77.2)	58/438 (13.2)	42/438 (9.6)
1-99	281/948 (29.6)	NA	186/281 (66.2)	95/281 (33.8)
≥100	229/948 (24.1)	NA	77/229 (33.6)	152/229 (66.4)

Abbreviations: CAC, coronary artery calcium; CCTA, coronary computed tomography angiography; LDL-C, low-density lipoprotein cholesterol; NA, not applicable.

^a^ Higher CAC scores indicate greater CAC burden.

The prevalence of any plaque (calcified or noncalcified) differed across LDL-C levels. Prevalence was 1363 (56.1%) for those with LDL-C levels of less than 77 mg/dL, 4099 (51.5%) for levels of 77 to 112 mg/dL, 4428 (52.7%) for levels of 113 to 154 mg/dL, 1935 (57.0%) for levels of 155 to 189 mg/dL, and 610 (64.3%) for levels of at least 190 mg/dL. 

The prevalence of CAC scores of 0 also differed across LDL-C levels, being lowest among 438 of 948 patients (46.2%) with LDL-C levels of at least 190 mg/dL and highest among 4370 of 7964 patients (54.9%) with LDL-C levels of 77 to 112 mg/dL and 4620 of 8409 (54.9%) patients with levels of 113 to 154 mg/dL (Table 2). In all LDL-C groups, a CAC score of 0 was associated with no coronary plaque in most patients (338 of 438 [77.2%] with LDL-C levels of ≥190 mg/dL and 1067 of 1204 [88.6%] with LDL-C levels of <77 mg/dL). However, the prevalence of noncalcified plaque increased stepwise among patients with CAC scores of 0 with increasing LDL-C levels, from 137 of 1204 patients (11.4%) with LDL-C levels of less than 77 mg/dL to 100 of 438 patients (22.8%) with LDL-C levels of at least 190 mg/dL (Table 2). The prevalence of obstructive plaque among patients with a CAC score of 0 also differed across LDL-C strata, being lowest among 56 of 1204 patients (4.7%) with LDL-C levels of less than 77 mg/dL and highest among 42 of 438 (9.6%) patients with LDL-C levels of at least 190 mg/dL.

The prevalence of calcified plaque had no clear association with LDL-C levels (Table 2). For example, the prevalence of CAC scores of at least 100 ranged from 1528 of 8409 patients (18.2%) with LDL-C levels of 113 to 154 mg/dL to 607 of 2430 patients (25.0%) with LDL-C levels of less than 77 mg/dL. Across all 3 CAC strata, however, the prevalence of obstructive coronary artery disease was highest in patients with LDL-C levels of at least 190 mg/dL. For example, the highest prevalence of obstructive coronary artery disease (152 of 229 [66.4%]) was found in patients with CAC scores of at least 100 and LDL-C levels of at least 190 mg/dL.

### Cardiovascular Disease Event Rates During Follow-up Stratified by CAC Burden and LDL-C Strata

**Figure 1.**
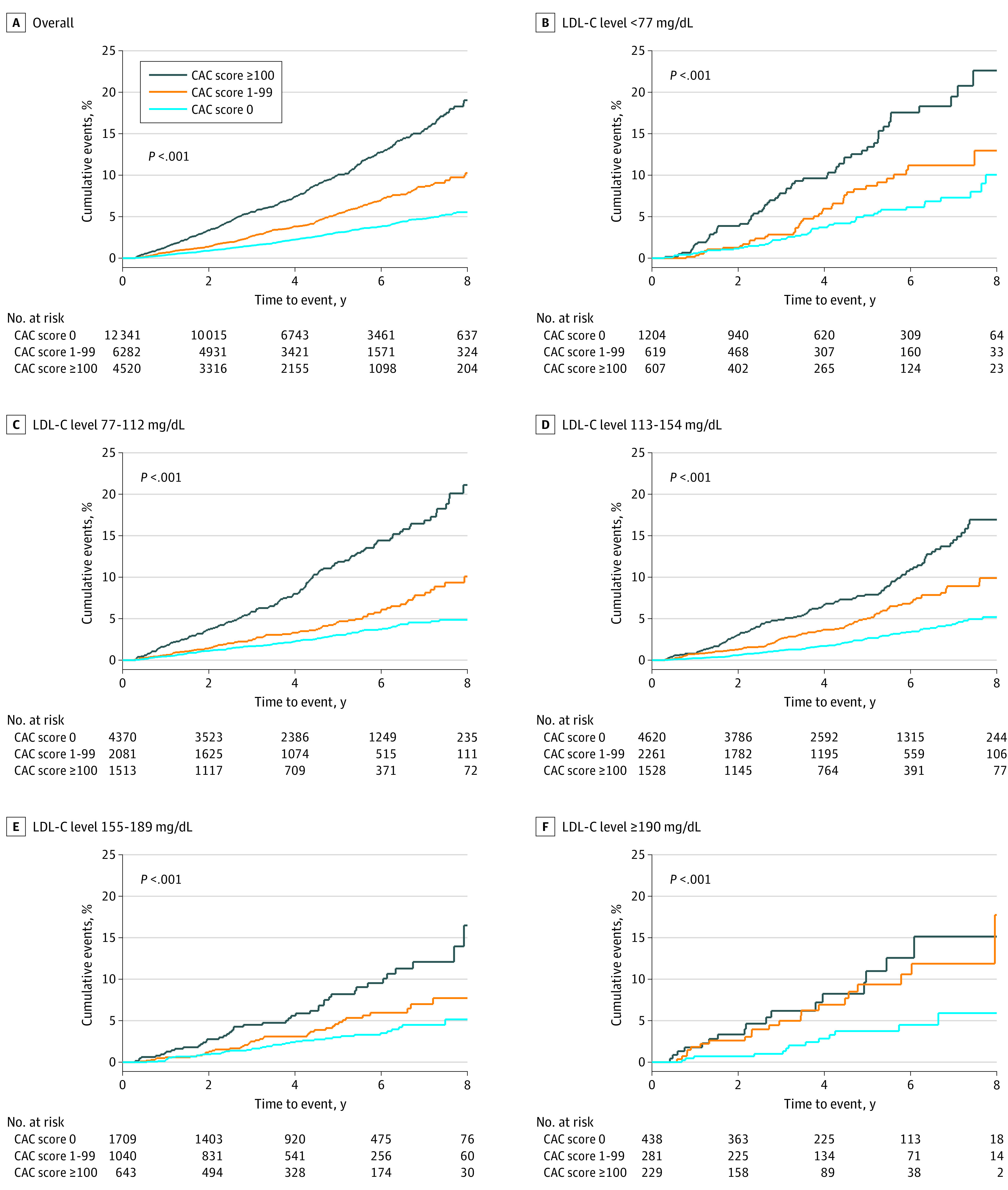
Cumulative Incidence of Cardiovascular Disease and Death Stratified by Coronary Artery Calcium (CAC) Burden and Low-Density Lipoprotein Cholesterol (LDL-C) Levels Higher CAC scores indicate greater CAC burden.

**Figure 2.**
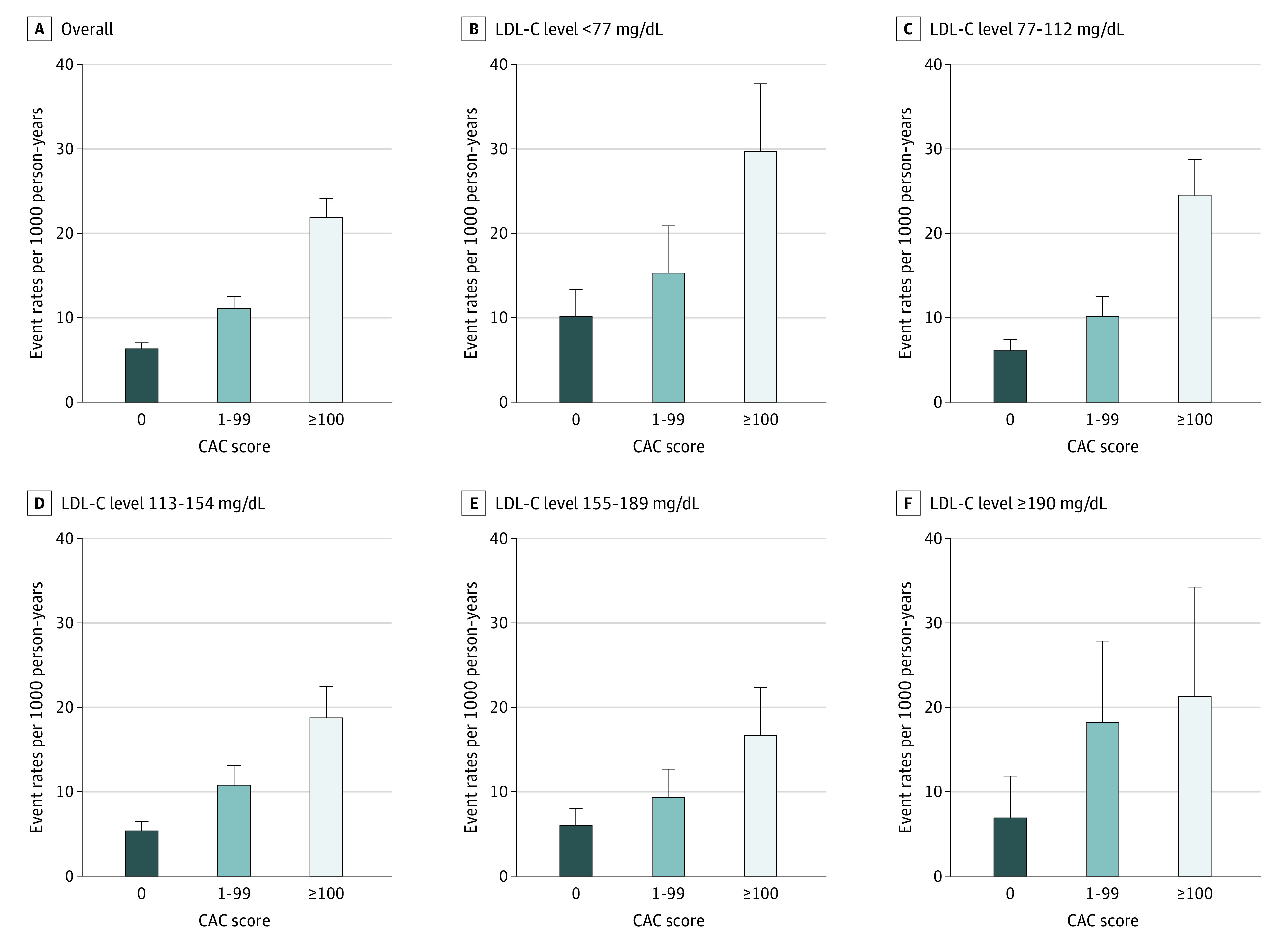
Event Rates of Cardiovascular Outcomes and All-Cause Death per 1000 Person-Years Stratified by Coronary Artery Calcium (CAC) Score and Low-Density Lipoprotein Cholesterol (LDL-C) Levels Higher CAC scores indicate greater CAC burden.

**Figure 3.**
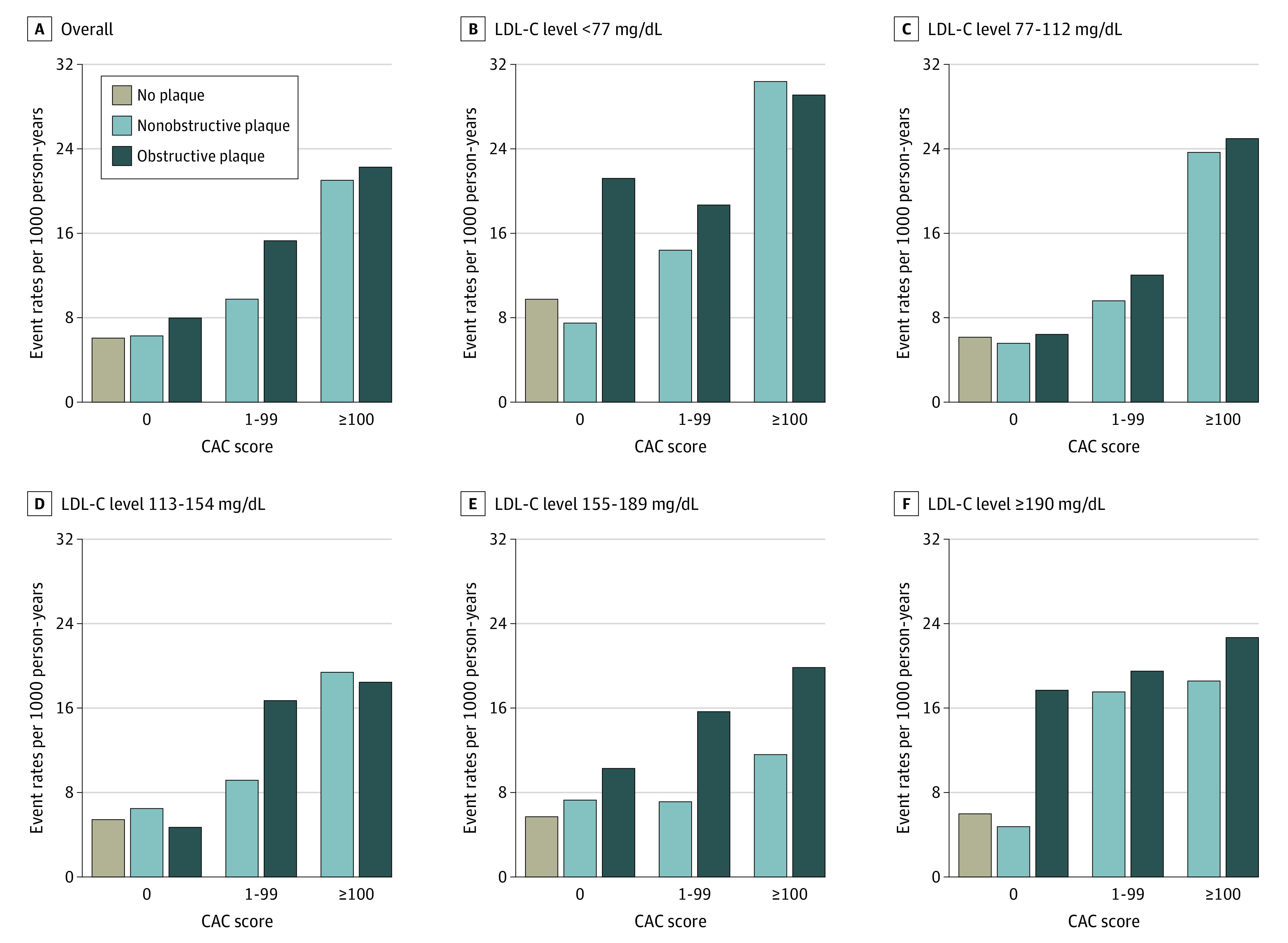
Event Rates of Cardiovascular Outcomes and All-Cause Deaths per 1000 Person-Years Stratified by Coronary Artery Calcium (CAC) Score, Presence of Nonobstructive or Obstructive Plaque, and Low-Density Lipoprotein Cholesterol (LDL-C) Levels Higher CAC scores indicate greater CAC burden.

Overall, absence of plaque was associated with low event rates across all LDL-C levels (6.3 [95% CI, 5.6-7.0] per 1000 person-years), with any detectable plaque (calcified or noncalcified) being associated with 2 to 3 times higher event rates (11.1 [95% CI, 10.0-12.5] and 21.9 [95% CI, 19.9-24.4] per 1000 person-years, respectively) (eFigure 1 [left] in the Supplement). For example, among patients with LDL-C levels of at least 190 mg/dL, the median event rate was 6.0 (95% CI, 3.1-11.5) in those with no plaque compared with 18.0 (95% CI, 18.4-24.4) in those with any plaque. Absence of CAC was also associated with low event rates across all LDL-C levels with stepwise increasing rates in patients with CAC scores of 1 to 99 and at least 100 (8-year cumulative incidence of approximately 5% in those with CAC scores of 0 compared with approximately 15%-20% in those with CAC scores of ≥100) (Figure 1). The overall median event rate in patients with CAC scores of 0 was 6.3 (95% CI, 5.6-7.0) compared with 10.2 (95% CI, 7.9-13.4) in those with LDL-C levels of less than 77 mg/dL and 6.9 (95% CI, 4.0-11.9) in those with LDL-C levels of at least 190 mg/dL (Figure 2). Among patients with CAC scores of 0, there was a tendency toward higher event rates in those with noncalcified plaque vs no plaque (event rates of 7.2 [95% CI, 5.3-9.4] vs 6.2 [95% CI, 5.4-7.0] per 1000 person-years; eFigure 1 [right] in the Supplement). This tendency was associated with higher event rates in those with CAC scores of 0 and presence of obstructive coronary artery disease (Figure 3), whereas event rates were comparable in those with CAC scores of 0 and no detectable plaque vs nonobstructive plaque (event rate among patients with LDL-C ≥190 mg/dL was 6.0 per 1000 person-years in patients with CAC scores of 0 and no plaque, 4.8 per 1000 person-years in patients with CAC socres of 0 and nonobstructive plaque, and 17.7 per 1000 person-years in patients with CAC scores of 0 and obstructive plaque) (Figure 3 and eTable 1 in the Supplement). In adjusted analyses, absence vs presence of CAC was associated with markedly lower risk for events in all LDL-C groups (eTable 2 in the Supplement). For example, the hazard ratio for CAC scores of 0 vs greater than 0 among those with LDL-C levels of at least 190 mg/dL was 0.49 (95% CI, 0.25-0.95).

### Sensitivity Analysis

In sensitivity analyses, we excluded all-cause death from the end point. As shown in eFigures 2 and 3 and eTable 3 in the Supplement, the results were similar to those of the main analyses, although event rates per 1000 person-years were lower in all groups.

In another sensitivity analysis, we excluded patients using statin therapy at baseline examination. As shown in eTable 4 in the Supplement, the overall results remained similar. Excluding patients younger than 30 years (n = 179) had no effect on the results.

## Discussion

The present study, based on a large contemporary cohort of consecutive symptomatic patients undergoing CCTA, provides important insights into the prevalence of noncalcified and calcified plaque and their associations with occurrence of cardiovascular disease across the LDL-C spectrum. To our knowledge, this study includes the largest published cohort to date of patients with LDL-C levels of at least 190 mg/dL. Four key points emerge from our analyses. First, atherosclerotic burden is heterogeneous across the spectrum of LDL-C levels, and risk is consistently associated with plaque burden. Second, we observed absence of plaque in 46.2% of patients with LDL-C levels of at least 190 mg/dL. This proportion was similar to that in patients with lower LDL-C levels. Third, CCTA-ascertained absence of CAC indicated no detectable plaque in 86.8% of patients, including those with LDL-C levels greater than 190 mg/dL. However, the prevalence of noncalcified plaque increased with higher LDL-C levels. Fourth, absence of plaque and CAC was associated with low event rates across the LDL-C spectrum, even when nonobstructive noncalcified plaques were present. Notably, however, when noncalcified obstructive coronary artery disease was present, event rates were high, demonstrating that CAC scores of 0 miss a small proportion of individuals at high risk.

Taken together, our results support the use of CCTA results for risk stratification (including derisking) of symptomatic patients with high LDL-C levels. This is important because such individuals are universally considered to be at high risk with very low LDL-C goals that can only be achieved by treatment with statins in combination with novel therapies to lower lipid levels. Among the large proportion of patients with LDL-C levels of at least 190 mg/dL who have no atherosclerotic plaque, the net benefit of such intensive treatment is questionable.

Advances in computed tomographic imaging have allowed for detailed description of coronary artery disease. Accordingly, CCTA testing is used increasingly in clinical practice, as endorsed by multiple guidelines.^1,2,16,17^ In addition to being able to identify obstructive coronary artery disease, CCTA provides in-depth information on atherosclerotic plaque composition in terms of both noncalcified and calcified plaque. Previous studies have shown that plaque burden is associated with future clinical events.^18,19,20,21,22^ In most studies, however, plaque burden has been assessed using CAC alone.^23,24,25^ Despite being a crude marker of atherosclerosis burden, CAC scores do not account for the burden of noncalcified plaque. Nevertheless, absence of CAC has been shown to be associated with low event rates in asymptomatic individuals, even in those who have multiple risk factors.^26,27,28^ Whether this also applies to symptomatic patients with high LDL-C levels has, however, been questioned owing to concern about a high prevalence of noncalcified plaque in such patients.^7^ Indeed, our study reveals a higher prevalence of noncalcified plaque in patients with severe elevation of LDL-C levels.

Patients with very high LDL-C levels (≥190 mg/dL) suggestive of possible clinical familial hypercholesterolemia are currently all considered to be at high risk, with strong recommendations for lifelong therapy to lower lipid levels in both US and European guidelines.^1,2,7^ The risk, however, is known to be heterogeneous.^29^ Even in untreated patients with genetically verified familial hypercholesterolemia, many do not develop clinical events.^30^ Further, within the same family, the clinical penetrance differs markedly. These findings demonstrate that a number of additional factors beyond elevated LDL-C levels affect atherogenesis in the individual patient, despite LDL-C level being the pathophysiological causal agent in atherogenesis.^31^ This principle is supported by the observation that ASCVD risk in patients with familial hypercholesterolemia is modified by other known risk factors beyond LDL-C levels.^7,31,32^ Thus, the theoretical advantage of using information about coronary artery disease severity from CCTA in patients with high LDL-C levels is its ability to provide insight into the lifetime exposure to both known and unknown risk modifiers as well as the susceptibility for developing atherosclerosis in the individual patients.^20^ Viewed in that context, our study provides important insights. Our data show that absence of atherosclerotic plaque and CAC in middle-aged symptomatic patients with high LDL-C levels (≥190 mg/dL) is a common finding associated with low risk. Even if nonobstructive, noncalcified plaque was present, absence of CAC was associated with low event rates. However, in individuals with obstructive noncalcified coronary artery disease, the event rate was substantially higher. Thus, if no obstructive coronary artery disease is present on the CCTA finding, the simple quantification of CAC can identify patients at low risk for ASCVD despite high LDL-C concentrations, without the need for more complex quantification of noncalcified plaque burden. This has important clinical implications because it suggests that allocation of add-on therapies to lower lipid levels, such as proprotein convertase subtilisin/kexin type 9 inhibitors, in patients with high LDL-C levels could be informed by severity of coronary artery disease. This information would allow for the allocation of expensive long-term therapies to those patients with severe hypercholesterolemia most likely to benefit, whereas such therapies could be avoided in patients with limited absolute benefit.^8^ That being said, the median follow-up in our study was relatively short at 4.2 years. Because 22.8% of patients with LDL-C levels of at least 190 mg/dL had detectable plaque despite having CAC scores of 0, the results should not be used to withhold long-term preventive therapy with statins, especially in younger individuals. Thus, the results may be used primarily to withhold additional novel and expensive therapies to lower lipid levels and thereby reach very low LDL-C level targets in those patients with CAC scores of 0 who would derive limited absolute benefit.

### Strengths and Limitations

Our study has several strengths. First, the data stem from a large all-comer real-world practice cohort. Second, in contrast to previous studies in asymptomatic cohorts that are criticized for potential healthy selection bias, our cohort is symptomatic and therefore at higher risk. The excellent prognosis in those with CAC scores of 0 in our population is therefore a reassuring finding. Third, to our knowledge, we included the largest sample to date of patients with LDL-C levels of at least 190 mg/dL undergoing CCTA. Fourth, no patients were lost to follow-up. Last, the results were robust in analyses using other end points or patient selection.

This study also has some limitations. First, our study population consisted of symptomatic patients. Therefore, the reported frequency of plaque may not be representative of that in asymptomatic individuals undergoing primary prevention. Nevertheless, because our study population would be expected to be at higher risk than a population undergoing purely primary prevention, absence of plaque is likely even more prevalent in the general population. Second, post-CCTA statin (and aspirin) use was high throughout the LDL-C spectrum. However, results remained similar when analyses were restricted to patients who did not use statin (or aspirin) after CCTA. Third, we did not quantify noncalcified plaque burden. Thus, it may be that quantification of noncalcified plaque volume may identify the few patients who despite having no CAC have extensive noncalcified plaque and are at high risk. Fourth, although all scans are analyzed by experienced cardiologists, there may be some misclassification of coronary artery disease. Finally, the median follow-up was 4.2 years, and it is unknown whether the results will change with longer follow-up.

## Conclusions

The findings of this cohort study suggest that among symptomatic patients with high LDL-C levels (≥190 mg/dL) who are considered at universally high risk for ASCVD in guidelines with low LDL-C goals, absence of calcified and noncalcified coronary plaque was associated with very low event rates. These results highlight the multifactorial character of atherosclerosis as a disease not only driven by LDL-C levels, despite their causal pathophysiological role. Our results also suggest that information on atherosclerotic plaque burden may be considered to individualize future treatment intensity and use of novel therapies to lower lipid levels in patients with severe hypercholesterolemia.

## Notes

Supplement. **eTable 1.** Event Rates per 1000 Person-Years of a Myocardial Infarction, Stroke, and All-Cause Death Stratified by Baseline LDL-C Levels, CAC Scores, and CCTA Plaque Burden **eTable 2.** Hazard Ratio for Having No Coronary Artery Calcium vs Having Coronary Artery Calcium Score >0 **eTable 3.** Event Rates per 1000 Person-Years of Myocardial Infarction and Stroke (Excluding All-Cause Death From the Study End Point), Overall and by Baseline LDL-C Levels, CAC Scores, and CCTA Plaque Burden **eTable 4.** Event Rates per 1000 Person-Years of Myocardial Infarction, Stroke, and All-Cause Death in Patients not Using Statin Therapy at Baseline Examination (Excluding Statin Users at Baseline), Overall and by Baseline LDL-C Levels, CAC Scores, and CCTA Plaque Burden **eFigure 1.** Cardiovascular and All-Cause Death Event Rates per 1000 Person-Years Stratified by Absence vs Presence of Detectable Atherosclerotic Plaque **eFigure 2.** Kaplan-Meier Cumulative Incidence Function for the Occurrence of Myocardial Infarction and Stroke (Excluding All-Cause Death From Study End Point) by Baseline LDL-C Levels and CAC Burden **eFigure 3.** Myocardial Infarction and Stroke Event Rates per 1000 Person-Years Stratified by Coronary Artery Calcium Score and Low-Density Lipoprotein Cholesterol Levels
