# Supplementary material for: Association of Coronary Plaque With Low-Density Lipoprotein Cholesterol Levels and Rates of Cardiovascular Disease Events Among Symptomatic Adults
Source: JAMA Netw Open. 2022 Feb 11;5(2):e2148139. doi: 10.1001/jamanetworkopen.2021.48139 (PMC8837910; doi:10.1001/jamanetworkopen.2021.48139)
Supplement: Supplement. — eTable 1. Event Rates per 1000 Person-Years of a Myocardial Infarction, Stroke, and All-Cause Death Stratified by Baseline LDL-C Levels, CAC Scores, and CCTA Plaque Burden eTable 2. Hazard Ratio for Having No Coronary Artery Calcium vs Having Coronary Artery Calcium Score >0 eTable 3. Event Rates per 1000 Person-Years of Myocardial Infarction and Stroke (Excluding All-Cause Death From the Study End Point), Overall and by Baseline LDL-C Levels, CAC Scores, and CCTA Plaque Burden eTable 4. Event Rates per 1000 Person-Years of Myocardial Infarction, Stroke, and All-Cause Death in Patients not Using Statin Therapy at Baseline Examination (Excluding Statin Users at Baseline), Overall and by Baseline LDL-C Levels, CAC Scores, and CCTA Plaque Burden eFigure 1. Cardiovascular and All-Cause Death Event Rates per 1000 Person-Years Stratified by Absence vs Presence of Detectable Atherosclerotic Plaque eFigure 2. Kaplan-Meier Cumulative Incidence Function for the Occurrence of Myocardial Infarction and Stroke (Excluding All-Cause Death From Study End Point) by Baseline LDL-C Levels and CAC Burden eFigure 3. Myocardial Infarction and Stroke Event Rates per 1000 Person-Years Stratified by Coronary Artery Calcium Score and Low-Density Lipoprotein Cholesterol Levels [file jamanetwopen-e2148139-s001.pdf]

Supplementary Online Content

Mortensen MB, Caínzos-Achirica M, Steffensen FH, et al. Association of coronary plaque with low-density lipoprotein cholesterol levels and rates of cardiovascular disease events among symptomatic adults. *JAMA Netw Open*. 2022;5(2):e2148139. doi:10.1001/jamanetworkopen.2021.48139

eTable 1. Event Rates per 1000 Person-Years of a Myocardial Infarction, Stroke, and All-Cause Death Stratified by Baseline LDL-C Levels, CAC Scores, and CCTA Plaque Burden

eTable 2. Hazard Ratio for Having No Coronary Artery Calcium vs Having Coronary Artery Calcium Score >0

eTable 3. Event Rates per 1000 Person-Years of Myocardial Infarction and Stroke (Excluding All-Cause Death From the Study End Point), Overall and by Baseline LDL-C Levels, CAC Scores, and CCTA Plaque Burden

eTable 4. Event Rates per 1000 Person-Years of Myocardial Infarction, Stroke, and All-Cause Death in Patients not Using Statin Therapy at Baseline Examination (Excluding Statin Users at Baseline), Overall and by Baseline LDL-C Levels, CAC Scores, and CCTA Plaque Burden

eFigure 1. Cardiovascular and All-Cause Death Event Rates per 1000 Person-Years Stratified by Absence vs Presence of Detectable Atherosclerotic Plaque

eFigure 2. Kaplan-Meier Cumulative Incidence Function for the Occurrence of Myocardial Infarction and Stroke (Excluding All-Cause Death From Study End Point) by Baseline LDL-C Levels and CAC Burden

eFigure 3. Myocardial Infarction and Stroke Event Rates per 1000 Person-Years Stratified by Coronary Artery Calcium Score and Low-Density Lipoprotein Cholesterol Levels

This supplementary material has been provided by the authors to give readers additional information about their work.

© 2022 Mortensen MB et al. *JAMA Network Open*.

eTable 1. Event Rates per 1000 Person-Years of a Myocardial Infarction, Stroke, and All-Cause Death Stratified by Baseline LDL-C Levels, CAC Scores, and CCTA Plaque Burden

	CCTA		
	**No plaque**	**Non obstructive**	**Obstructive**
**Overall (N=23,143)**			
CAC=0	6.1 (5.5-6.9)	6.3 (4.1-9.9)	8.0 (5.3-11.8)
CAC 1-99	-	9.8 (8.5-11.3)	15.3 (12.6-18.8)
CAC ≥100	-	21.1 (18.0-24.7)	22.3 (19.7-25.4)
**LDL-C <2.0 (N=2,430)**			
CAC=0	9.8 (7.3-13.1)	7.5 (1.9-31.0)	21.2 (8.8-51.0)
CAC 1-99	-	14.4 (10.0-20.7)	18.7 (10.0-34.7)
CAC ≥100	-	30.4 (21.3-43.5)	29.1 (21.1-40.2)
**LDL-C 2.0-2.9 (N=7,964)**			
CAC=0	6.2 (5.1-7.5)	5.6 (2.3-13.5)	6.4 (3.1-13.5)
CAC 1-99	-	9.6 (7.5-12.2)	12.1 (8.2-18.0)
CAC ≥100	-	23.7 (18.3-30.6)	25.0 (20.3-30.7)
**LDL-C 3.0-3.9 (N=8,409)**			
CAC=0	5.4 (4.4-6.6)	6.5 (3.2-12.9)	4.7 (2.1-10.6)
CAC 1-99	-	9.2 (7.2-11.6)	16.7 (12.0-23.2)
CAC ≥100	-	19.4 (14.7-25.5)	18.5 (14.5-23.4)
**LDL-C 4.0-4.9 (N=3,392)**			
CAC=0	5.7 (4.1-7.8)	7.3 (2.7-19.4)	10.3 (3.9-27.6)
CAC 1-99	-	7.1 (4.7-10.6)	15.7 (9.9-24.9)

© 2022 Mortensen MB et al. *JAMA Network Open*.

CAC ≥100	-	11.6 (6.6-20.4)	19.9 (14.1-28.0)
**LDL-C ≥5 (N=948)**			
CAC=0	6.0 (3.1-11.6)	4.8 (0.7-34.2)	17.7 (5.7-54.9)
CAC 1-99	-	17.5 (10.2-30.1)	19.5 (9.7-38.9)
CAC ≥100	-	18.6 (7.7-44.6)	22.7 (12.9-40.0)

The main study endpoint combined myocardial infarction, ischemic stroke and all-cause death. Results are presented as incidence rates (per 1,000 person-years)
Abbreviations: CAC = coronary artery calcium; CCTA = coronary computed tomography angiography; LDL-C = low-density lipoprotein cholesterol

© 2022 Mortensen MB et al. *JAMA Network Open*.

eTable 2. Hazard Ratio for Having No Coronary Artery Calcium vs Having Coronary Artery Calcium Score >0

	Hazard ratio for cardiovascular disease and death (95% CI)*					
	**Overall**	**LDL<2**	**LDL 2.0-2.9**	**LDL 3.0-3.9**	**LDL 4.0-4.9**	**LDL≥5.0**
**CAC 0 vs. CAC >0**	0.74 (0.63-0.90)	0.78 (0.52-1.14)	0.78 (0.64-0.99)	0.67 (0.54-0.93)	0.82 (0.61-1.05)	0.49 (0.25-0.95)

*Adjusted by age, sex, smoking status, statin use at baseline, aspirin use at baseline and post-CCTA statin use.

© 2022 Mortensen MB et al. *JAMA Network Open*.

eTable 3. Event Rates per 1000 Person-YEARS of Myocardial Infarction and Stroke (Excluding All-Cause Death From the Study End Point), Overall and by Baseline LDL-C Levels, CAC Scores, and CCTA Plaque Burden

	**CCTA**		
	**No plaque**	**Non obstructive**	**Obstructive**
**Overall (N=23,143)**			
CAC=0	3.3 (2.8-3.8)	4.5 (2.6-7.5)	4.5 (2.6-7.5)
CAC 1-99	-	4.4 (3.5-5.4)	9.6 (7.4-12.4)
CAC >100	-	8.8 (6.9-11.2)	11.2 (9.3-13.4)
**LDL-C <2.0 (N=2,430)**			
CAC=0	4.8 (3.2-7.3)	7.8 (1.9-31.0)	8.5 (2.1-33.9)
CAC 1-99	-	6.0 (3.4-10.5)	11.2 (5.0-24.9)
CAC >100	-	10.1 (5.5-18.8)	14.2 (8.9-22.5)
**LDL-C 2.0-2.9 (N=7,964)**			
CAC=0	3.2 (2.5-4.2)	5.6 (2.3-13.5)	2.8 (0.9-8.6)
CAC 1-99	-	4.3 (3.0-6.2)	6.8 (4.0-11.5)
CAC >100	-	9.3 (6.2-14.2)	12.2 (9.1-16.4)
**LDL-C 3.0-3.9 (N=8,409)**			
CAC=0	3.2 (2.4-4.1)	4.0 (1.7-9.7)	3.2 (1.2-8.4)
CAC 1-99	-	3.5 (2.4-5.1)	9.5 (6.1-14.8)
CAC >100	-	8.7 (5.8-13.1)	8.7 (6.1-12.3)
**LDL-C 4.0-4.9 (N=3,392)**			
CAC=0	2.5 (1.5-4.0)	3.5 (0.9-13.8)	7.4 (2.4-22.9)
CAC 1-99	-	3.9 (2.3-6.7)	11.6 (6.9-19.6)
CAC >100	-	3.8 (1.4-10.1)	12.1 (7.9-18.6)

© 2022 Mortensen MB et al. *JAMA Network Open*.

**LDL-C ≥5 (N=948)**			
CAC=0	4.0 (1.8-8.9)	0	11.8 (3.0-47.2)
CAC 1-99	-	12.1 (6.3-23.3)	14.6 (6.5-32.5)
CAC >100	-	18.6 (7.7-44.6)	11.3 (5.1-25.3)

The secondary study endpoint included myocardial infarction and ischemic stroke. Results are presented as incidence rates (per 1,000 person-years)
Abbreviations: CAC = coronary artery calcium; CCTA = coronary computed tomography angiography; LDL-C = low-density lipoprotein cholesterol

© 2022 Mortensen MB et al. *JAMA Network Open*.

eTable 4. Event Rates per 1000 Person-Years of Myocardial Infarction, Stroke, and All-Cause Death in Patients not Using Statin Therapy at Baseline Examination (Excluding Statin Users at Baseline), Overall and by Baseline LDL-C Levels, CAC Scores, and CCTA Plaque Burden

	**CCTA**		
	**No plaque**	**Non obstructive**	**Obstructive**
**Overall (N=23,143)**			
CAC=0	5.7 (4.9-6.5)	6.0 (3.4-10.5)	3.9 (1.9-7.6)
CAC 1-99	-	8.7 (6.7-11.3)	15.3 (12.6-18.8)
CAC ≥100	-	31.2 (19.7-49.6)	29.11 (21.1-40.1)
**LDL-C <2.0 (N=2,430)**			
CAC=0	9.1 (5.9-13.9)	23.7 (5.9-95.2)	18.2 (2.6-129.6)
CAC 1-99	-	12.4 (6.2-24.8)	18.7 (10.0-34.7)
CAC ≥100	-	30.4 (21.3-43.5)	29.1 (20.3-40.7)
**LDL-C 2.0-2.9 (N=7,964)**			
CAC=0	5.6 (4.4-7.0)	3.9 (0.9-15.7)	2.7 (0.7-10.9)
CAC 1-99	-	9.0 (5.0-12.6)	12.1 (8.2-18.0)
CAC ≥100	-	24.1 (17.5-33.3)	25.0 (20.3-30.7)
**LDL-C 3.0-3.9 (N=8,409)**			
CAC=0	5.4 (4.3-6.6)	6.3 (2.8-13.9)	3.1 (1.0-9.4)
CAC 1-99	-	8.6 (5.5-13.2)	16.6 (12.0-23.2)
CAC ≥100	-	19.3 (13.7-27.3)	18.4 (14.5-23.4)
**LDL-C 4.0-4.9 (N=3,392)**			
CAC=0	5.7 (3.9-8.3)	3.0 (0.4-19.4)	4.2 (0.6-29.6)
CAC 1-99	-	6.9 (3.5-13.9)	15.7 (9.8-24.7)

© 2022 Mortensen MB et al. *JAMA Network Open*.

CAC ≥100	-	9.2 (3.8-22.1)	19.9 (13.2-28.0)
**LDL-C ≥5 (N=948)**			
CAC=0	4.1 (4.1-12.6)	5.0 (1.7-85.1)	13.5 (1.9-96.7)
CAC 1-99	-	17.7 (6.6-42.7)	19.5 (9.7-38.9)
CAC ≥100	-	24.2 (9.1-64.3)	22.4 (12-1-40.0)

The secondary study endpoint included myocardial infarction and ischemic stroke. Results are presented as incidence rates (per 1,000 person-years)
Abbreviations: CAC = coronary artery calcium; CCTA = coronary computed tomography angiography; LDL-C = low-density lipoprotein cholesterol.

© 2022 Mortensen MB et al. *JAMA Network Open*.

eFigure 1. Cardiovascular and All-Cause Death Event Rates per 1000 Person-Years Stratified by Absence vs Presence of Detectable Atherosclerotic Plaque

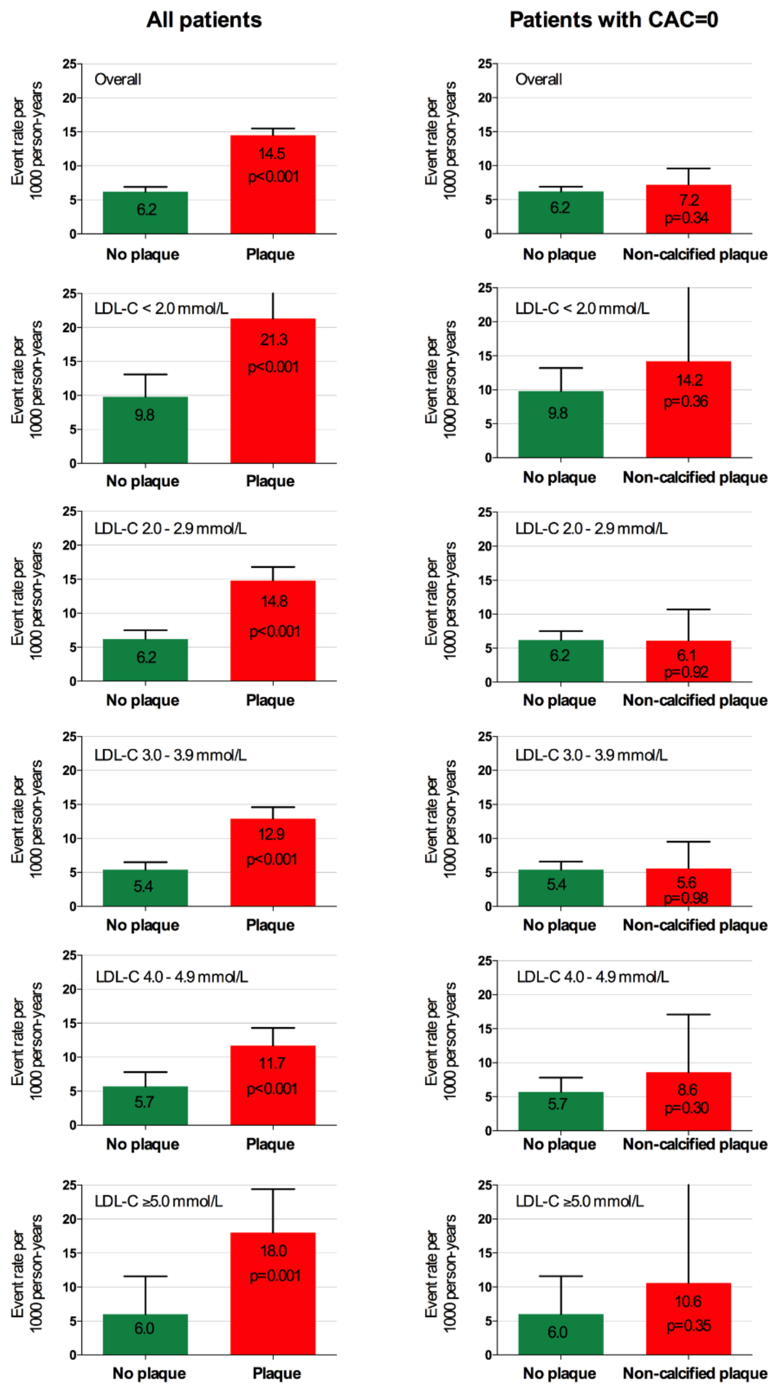

© 2022 Mortensen MB et al. *JAMA Network Open*.

eFigure 2. Kaplan-Meier Cumulative Incidence Function for the Occurrence of Myocardial Infarction and Stroke (Excluding All-Cause Death From Study End Point) by Baseline LDL-C Levels and CAC Burden

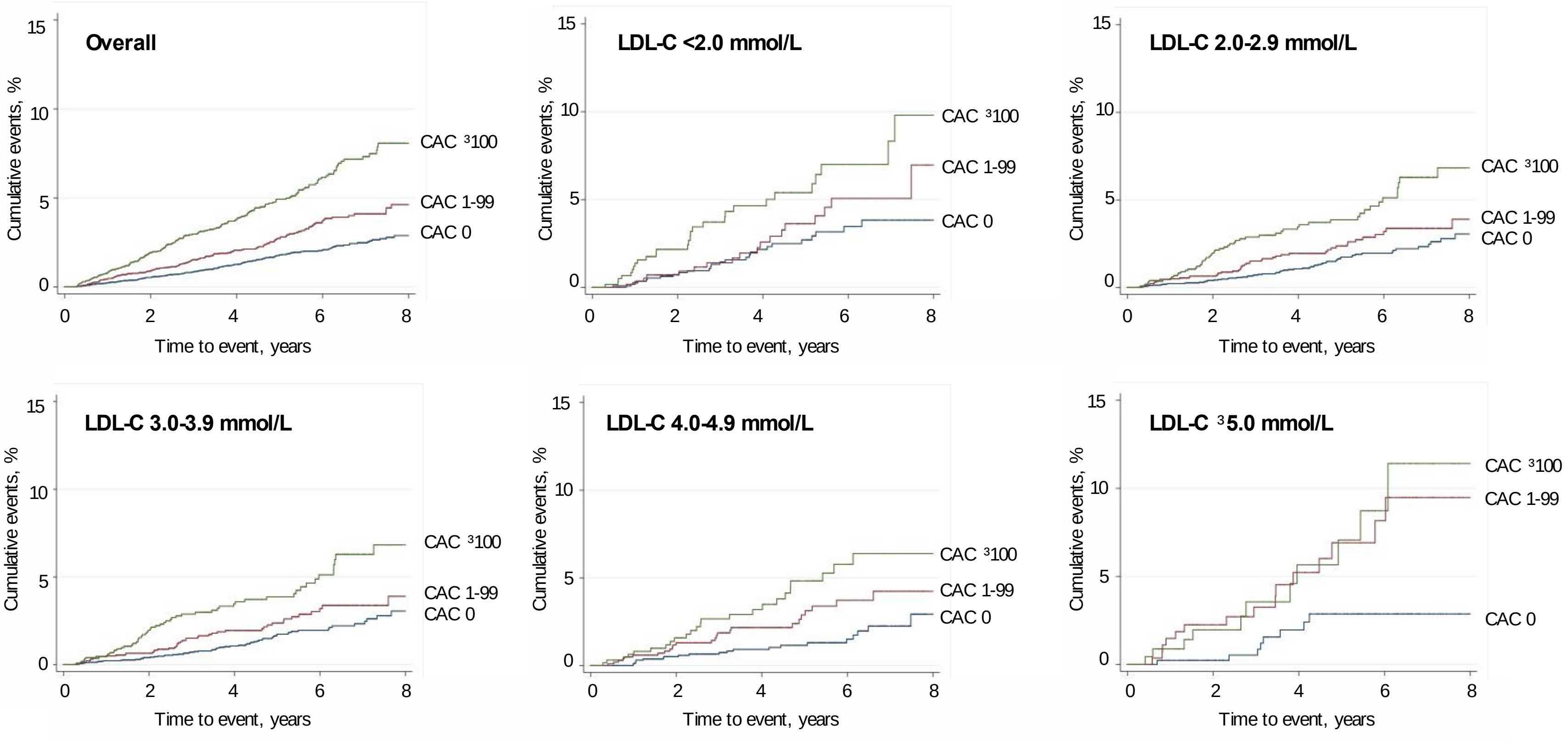

The secondary study endpoint included myocardial infarction and ischemic stroke.

Abbreviations: CAC = coronary artery calcium; LDL-C = low-density lipoprotein cholesterol

© 2022 Mortensen MB et al. *JAMA Network Open*.

eFigure 3. Myocardial Infarction and Stroke Event Rates per 1000 Person-Years Stratified by Coronary Artery Calcium Score and Low-Density Lipoprotein Cholesterol Levels

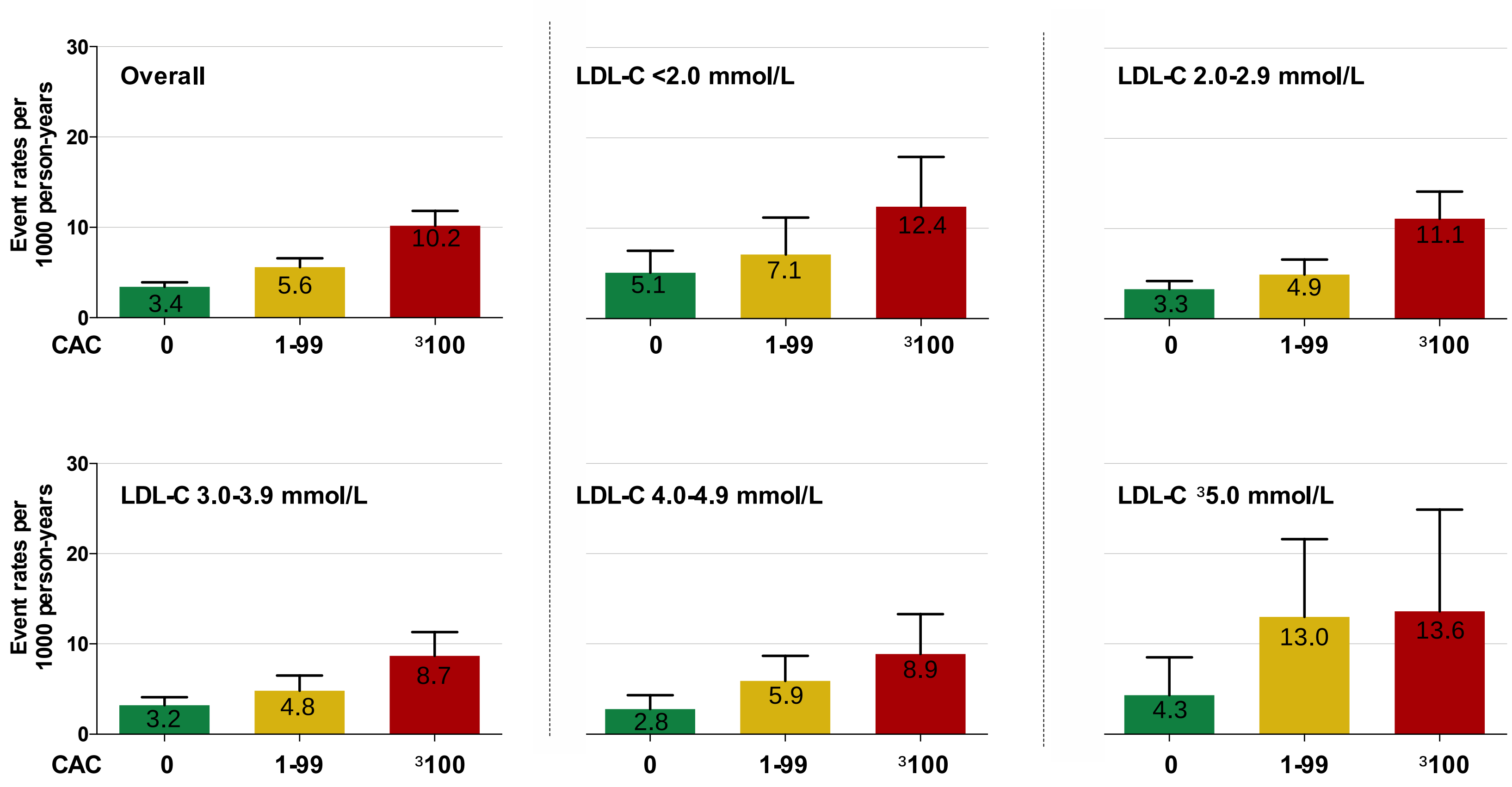

© 2022 Mortensen MB et al. *JAMA Network Open*.
